# Damaged DNA marching out of aging nucleus

**DOI:** 10.18632/aging.102340

**Published:** 2019-10-02

**Authors:** Nir Hacohen, Yuk Yuen Lan

**Affiliations:** 1 Center for Cancer Research, Massachusetts General Hospital, Charlestown, MA 02114, USA; 2 Department of Medicine, Harvard Medical School, Boston, MA 02115, USA

**Keywords:** DNASE2, DNA sensing, inflammation, cellular senescence, STING

Subclinical but heightened inflammation in the absence of infection is a key feature of aging, and includes senescent cells that secrete cytokines. Yet, what are the intrinsic processes that initiate ‘inflammaging’, and possibly other forms of sterile inflammation, like autoimmunity?

**Figure 1:**
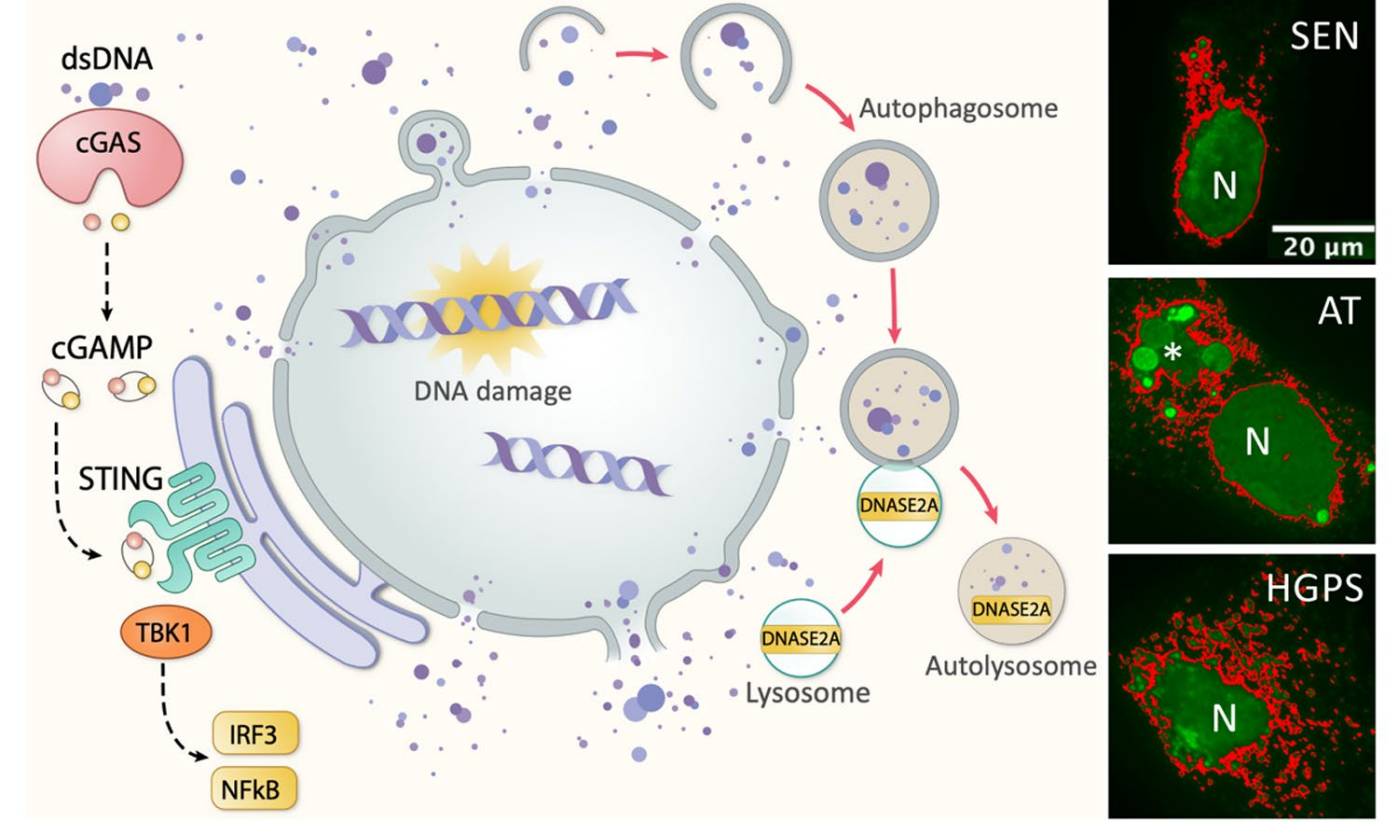
**Damaged nuclear DNA export, sensing and clearance in aging-related inflammation.** Left, schematic showing nuclear-autophagy-lysosome DNA clearance pathway in which nuclear DNA trafficked to cytosol is enclosed by autophagosomes and delivered to lysosomes for degradation by DNASE2A. Excess extranuclear DNA accumulated upon increased DNA damage (old cells), deficit repair (AT), deformed nuclear barrier (HGPS), or defective degradation (Dnase2a-/-), can activate innate DNA sensing cGAS-cGAMP-STING pathway and induce aging-associated IFN response and inflammation. Right, immunofluorescent images of anti-dsDNA staining (green) in replicative senescent (SEN), AT and HGPS human fibroblasts. Pseudo-colored (red) overlaid to enhance visualization of lobulated nuclear envelop and excess extranuclear DNA burden in forms of buds, speckles and large aggregates (asterisk in AT); N, nucleus.

Self-DNA has long been suspected as trigger and target of autoimmunity, as anti-nuclear antibodies, anti-dsDNA (double-stranded DNA) antibodies and plasma DNA are observed in autoimmune patients of lupus and rheumatoid arthritis. In studying the initiating events leading to autoimmune arthritis in mice deficient for the lysosomal nuclease DNASE2A, we revealed an unexpected ‘hidden’ source of this inflammatory DNA—the cell’s own nucleus [1]. We discovered a cell autonomous nuclear-to-lysosome pathway that removes immunogenic self-DNA. In healthy cells, damaged and irreparable nuclear DNA fragments are trafficked to the cytosol, enclosed by autophagosomes, and delivered to the lysosomes for degradation by DNASE2A. Lacking DNASE2A, extranuclear DNA accumulates in cells and induces inflammation via innate DNA sensing. Cytosolic DNA sensing is activated when dsDNA binds the DNA sensor enzyme cGAS (cyclic GMP-AMP synthase), converting GTP and ATP into the endogenous second messenger cGAMP, which in turns activates the adaptor protein STING (stimulator of interferon genes) and induces innate immune responses and inflammation (see Figure 1). Nuclear DNA as a trigger of immunity could help explain a range of inflammatory conditions.

As cells age, damaged DNA accumulates over time. As an interesting aside, anti-dsDNA antibodies are also found at higher levels in older adults [2]. Could damaged DNA march out of the nucleus of an old cell to set off inflammaging? Indeed, in replicative and oncogene-induced senescent cells, damaged nuclear DNA is exported in the form of nuclear buds, cytosolic speckles or fragments [3,4], with nuclear DNA blebs recognized by the DNA sensor cGAS [5]. Excess DNA in old cells triggers the cGAS-STING axis enhancing type I interferon and IL-6 signaling [4]; and regulates a later program of paracrine SASP [3,5,6]. Through the same mechanism, intrinsic DNA burden caused by deficient DNA repair or leaky nuclear envelope in cells from patients with the aging diseases ataxia telangiectasia (AT) or Hutchinson-Gilford progeria (HGPS) (see IF images) also mounts an innate immune activation and STING-dependent p16 expression [4]. Elevated cytosolic load of intrinsic DNA contributes to persistent inflammation in aging-related conditions.

Clearing DNA is perhaps the most effective way to eliminate its inflammatory danger. As the only known acidic DNA endonuclease, DNASE2A preferentially degrades dsDNA. It resides with the lysosome, where intracellular and extracellular DNA cargoes converge for degradative digestion. Facilitated by autophagic transport or active engulfment, DNASE2A functions cell-autonomously to degrade damaged nuclear DNA, pyknotic nuclei from erythrocytes and apoptotic DNA fragments. In humans, biallelic loss-of-function mutation in DNASE2A results in type I interferonopathy with increased anti-DNA antibodies [7]. In mice, Dnase2a-deficient cells exhibits the typical senescent phenotype of enlarged cells, slow cell growth and increased expression of aging markers (senescence-associated β-gal activity, p16 and HP1β expression) [4]. Indeed, ectopic expression of DNASE2A substantially reduces cytosolic DNA abundance, innate immune activation and cellular aging phenotype in old cells [4,8], thus confirming the protective role of enzymatic DNA degradation in limiting inflammation.

Growing evidence now supports a unifying theory that damaged or irreparable DNA leaves the nucleus to drive aging-related inflammation via innate DNA sensing. Where DNA damage is increased (aging), DNA repair inhibited (ataxia), or nuclear barrier compromised (progeria), DNA load may be not reduced promptly or sufficiently, leading to inflammation. So how far can this DNA theory help to understand the cellular immune mechanisms underlying aging? Each nucleus holds a massive reservoir of endogenous DNA that can trigger local and systemic immunity if there are internal abnormalities such as DNA damage. How nuclear DNA export, trafficking, sensing and degradation is coordinated to maintain cellular homeostasis is largely unknown. DNA danger coming from within generates exciting questions that probe into the basic life cycle of broken DNA fragments, and suggest ways of treating self-DNA-mediated sterile inflammation (autoimmunity, cancer, neurodegeneration and chemotherapy) by regulating the abundance of mis-localized DNA.
